# Assessing the relationship between multimorbidity, NCD configurations, frailty phenotypes, and mortality risk in older adults

**DOI:** 10.1186/s12877-024-04948-9

**Published:** 2024-04-22

**Authors:** Rafael Ogaz-González, Eva Corpeleijn, Rosa Estela García-Chanes, Luis Miguel Gutierréz-Robledo, Ricardo Antonio Escamilla-Santiago, Malaquías López-Cervantes

**Affiliations:** 1 Department of Public Health, Faculty of Medicine, National Autonomous University of México, Sixth Floor, Building B, 411A Circuito Escolar, Copilco Universidad, Mexico City, Coyoacán 04360 Mexico; 2 Department of Epidemiology, University of Groningen, University Medical Center Groningen, Groningen, The Netherlands; 3 Dirección de Investigación, Instituto Nacional de Geriatría, Ciudad de México, Mexico

**Keywords:** Multimorbidity, Patterns, Clusters, Frailty, Mortality, Older adults

## Abstract

**Background:**

Older adults are increasingly susceptible to prolonged illness, multiple chronic diseases, and disabilities, which can lead to the coexistence of multimorbidity and frailty. Multimorbidity may result in various noncommunicable disease (NCD) patterns or configurations that could be associated with frailty and death. Mortality risk may vary depending on the presence of specific chronic diseases configurations or frailty.

**Methods:**

The aim was to examine the impact of NCD configurations on mortality risk among older adults with distinct frailty phenotypes. The population was analyzed from the Costa Rican Longevity and Healthy Aging Study Cohort (CRELES). A total of 2,662 adults aged 60 or older were included and followed for 5 years. Exploratory factor analysis and various clustering techniques were utilized to identify NCD configurations. The frequency of NCD accumulation was also assessed for a multimorbidity definition. Frailty phenotypes were set according to Fried et al. criteria. Kaplan‒Meier survival analyses, mortality rates, and Cox proportional hazards models were estimated.

**Results:**

Four different types of patterns were identified: ‘Neuro-psychiatric’, ‘Metabolic’, ‘Cardiovascular’, and ‘Mixt’ configurations. These configurations showed a higher mortality risk than the mere accumulation of NCDs [Cardiovascular HR:1.65 (1.07–2.57); ‘Mixt’ HR:1.49 (1.00-2.22); ≥3 NCDs HR:1.31 (1.09–1.58)]. Frailty exhibited a high and constant mortality risk, irrespective of the presence of any NCD configuration or multimorbidity definition. However, HRs decreased and lost statistical significance when phenotypes were considered in the Cox models [frailty + ‘Cardiovascular’ HR:1.56 (1.00-2.42); frailty + ‘Mixt’:1.42 (0.95–2.11); and frailty + ≥ 3 NCDs HR:1.23 (1.02–1.49)].

**Conclusions:**

Frailty accompanying multimorbidity emerges as a more crucial indicator of mortality risk than multimorbidity alone. Therefore, studying NCD configurations is worthwhile as they may offer improved risk profiles for mortality as alternatives to straightforward counts.

**Supplementary Information:**

The online version contains supplementary material available at 10.1186/s12877-024-04948-9.

## Background

As individuals age, their physiological responses tend to weaken [1, 2]. More importantly, the number of years in good health has not progressed at the same pace as for life expectancy. Aging is a complex process characterized by numerous physiological and functional declines, which in turn escalate the risk of adverse health outcomes, including mortality [3, 4]. This decline in bodily functions contributes to the accumulation of syndromes and diseases, ultimately leading to a state of multimorbidity (MM) [5–7] or frailty [8]. Very often, MM goes along with frailty. Frailty is a consequence of multiple deficiencies in systems and a state of vulnerability characterized by disabilities and poor resolution to physiologic stress [9]. Although frailty has been mainly related to cellular senescence, oxidative stress, and cellular damage, new evidence points in the direction of MM to understand the bridge between health and frailty [10].

The precise definition of MM remains subject to debate [11–13]; for example, it is understood as the presence of two or more chronic diseases or as any coexisting diseases [14]. Nevertheless, one of the main difficulties of this scope is the possibility of numerous combinations for grouping the diseases [11]. Efforts have been made to condense multimorbidity into distinct patterns, yielding varied outcomes that are often perceived as random. However, these patterns may be indicative of grouping based on risk factors or demographic characteristics. Some authors emphasize the crucial task of identifying and customizing these coexistence patterns and evaluating their relationship with specific outcomes [11–13, 15, 16].

Scientific evidence suggests that 37.2% of the global population has multimorbidity [17]. This prevalence tends to increase with age, affecting approximately 65% of individuals aged 65 to 84 years and rising to 82% among those aged 85 years and older. Although around 70% of all deaths worldwide had been attributable to non-communicable diseases (NCDs) [18, 19], certain aggregations of NCD may have more negative impact on quality of life [20], increase the demand for specific health care services [21] and pose an even greater mortality risk [22] than others. In addition, multimorbidity has been related to accelerated ageing and to frailty [10, 23, 24], which in turn, has an impact on the burden of health systems and affects quality of ageing [25].

To define frailty, several countries and organizations have adopted the approach outlined by the World Health Organization (WHO), as follows: “*a progressive age-related decline in physiological systems that results in decreased reserves of intrinsic capacity, which confers extreme vulnerability to stressors and increases the risk of a range of adverse health outcomes*” [26]. Frailty prevalence also relates to aging, ranging from 12% among 60-69-year-old adults to 46% for those aged 90 [27], however, it is thought that the conformation of specific NCD patterns over age may contribute to this phenomenon [28, 29]. Healthcare systems face significant challenges in caring for older individuals [30]. The special needs and extended treatment regimens in this population underscores the importance of accurately identifying the root causes of health issues. Some authors suggest that frailty may have a more profound impact on disabilities and mortality than multimorbidity [31]. However, the debate calls for a broader perspective, acknowledging that health is a multidimensional phenomenon rather than to follow an independent line of causation [32, 33].

The risk of mortality in older adults varies based on the frailty phenotype and is influenced by specific diseases. However, this risk appears to differ when considering the cumulative effect of chronic diseases [32]. The gap in knowledge about the relationship of multimorbidity patterns and frailty is still not well understood, and there might be an influence on how different configurations in accumulation of NCDs can influence mortality in older adults with distinct phenotypes of frailty.

The aim of this study is to examine the impact of multimorbidity and different NCDs configurations on mortality risk among older adults with distinct frailty phenotypes.

## Methods

### Data and participants

The study is a prospective analysis from the publicly available Costa Rican Longevity and Healthy Aging Study Cohort (CRELES),, which focused on investigating healthy aging in older adults born in 1945 or earlier in Costa Rica. The CRELES cohort comprised a nationally representative sample of 2,827 individuals aged 60 years or older was selected through a stratified probabilistic sampling design, with an over-sample of people aged 95 and over. Data collection occurred through three waves of household standardized interviews conducted by specialized personnel. The follow-up intervals between waves were approximately 2 years. CRELES encompasses information on participants’ diverse aspects, including self-reported physical and psychological health, living conditions, health behaviors, health care utilization, social support, and socioeconomic status. Additionally, it includes health indicators like anthropometry, mobility, and biomarkers. Mortality events were monitored through linkages with the Costa Rican National Death Index, and details surrounding death were acquired through interviews with surviving family members. A detailed account of sampling methods, data collection, and objectives can be found elsewhere [34].

**Fig. 1:**
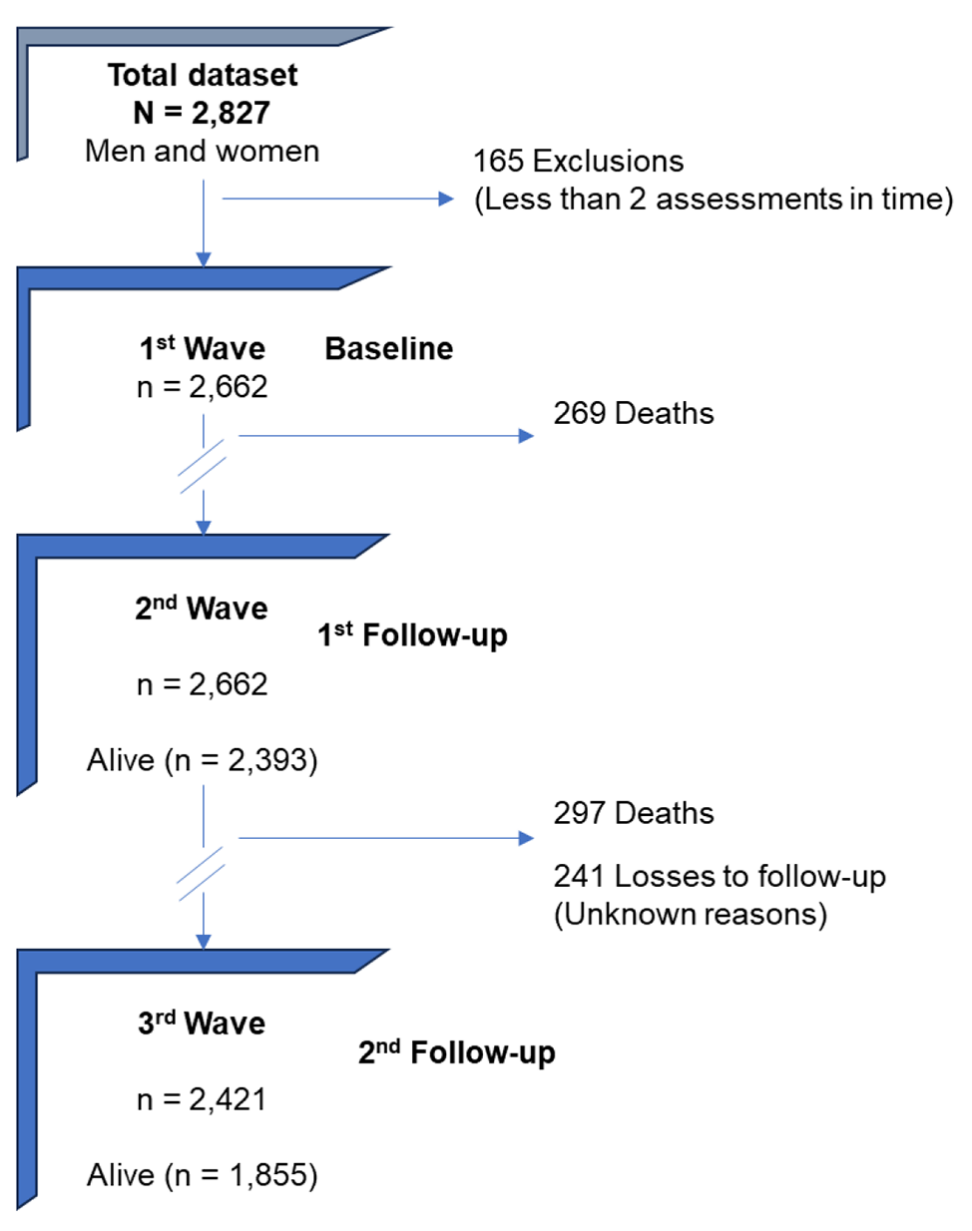
Cohort follow-up procession: exclusion, inclusion, and follow-up of subjects with phenotype criteria throughout the time in the CRELES Cohort

To be included in the analysis, the criteria required at least two assessments over the entire follow-up period. Out of the initial 2,827 participants in the CRELES cohort, 165 subjects (5.8%) did not meet the inclusion criteria at baseline (1st wave). All subjects that were excluded from the analysis were individuals with only one assessment between the first and second waves, so there was no losses to follow-up between the baseline and the first follow-up (2nd wave), but 241 subjects (9.1%) were lost between the first and second follow-up (3rd wave). At the conclusion of the assessments, 1,855 older individuals were still alive, while 566 (21.3%) had passed away. This resulted in a final dataset of 2,421 observations for the analysis (Fig. 1).

### Analysis

To determine MM and NCDs configurations, two approaches were adopted. First, we identified those individuals without any NCD to conform a group of subjects with No NCDs, then we calculated the NCD frequency by adding up the number of chronic diseases at baseline. MM by accumulation was defined as having at least three chronic diseases because in this population, this distribution relates to mortality more than the WHO definition of 2 or more. Second, we used an exploratory factor analysis (EFA) to identify NCD configurations at baseline based on presence/absence of the NCDs and operating under the assumption that some diseases co-occur selectively with other diseases.

To determine NCD presence, we used structured questions from questionnaires to ascertain the diagnosis of a disease and medication use for each NCD. If in the questionnaires “Yes” had been answered to a question about previous diagnosis from a physician, then the referred disease was categorized as having it (e.g., Has a physician ever told you that you have high blood pressure?). Responding “Yes” for using medication to control a disease (e.g., Are you taking insulin injections to control your diabetes?) Selecting a medication from another specific structured instrument was the procedure used to evaluate medication consumption.

The following list of 13 diseases were considered NCDs: Parkinson’s disease, chronic nervous problems (being diagnosed with a nervous or psychiatric problem such as depression), chronic respiratory or pulmonary diseases (including emphysema, tuberculosis, asthma, or chronic bronchitis), arthritis (including rheumatism or arthrosis), osteoporosis, stroke (including the answer for how many strokes have you had in your life?), heart attack (included the answer for how many heart attacks have you had in your life?), other heart diseases (without having a heart attack), and cancer (including any cancer or a malignant tumor, but not small skin tumors) Questionnaire report assessments for cancer also included selecting which organ or part of the body cancer began (stomach, other digestive, urinary, leukemia, lung, other respiratory, prostate, uterine/cervical, mammary, and other). Hypertension (HTA) was additionally defined as having a systolic/diastolic pressure ≥ 140/90, diabetes (DM) as the levels of glycosylated hemoglobin (HbA1c ≥ 6.5%) in collected blood samples [35, 36], obesity as having a body mass index (BMI) ≥ 30 kg/m2) [37], and depression as assessed with the short version of the Geriatric Depression Scale [38, 39].

Frailty was assessed as phenotypes using a five-component accumulation scale, as proposed by L. Fried et al. [40]. Two criteria from the L. Fried scale were adapted to the available data: poor endurance and energy (self-reported exhaustion in the last week) [38], and low physical activity level [39].

To evaluate unintentional weight loss, participants were asked, “Have you experienced unintentional weight loss exceeding 5 kilograms in the last 6 months? (Yes/No)” To assess fatigue/exhaustion, participants were asked, “Did you feel full of energy during the past week? (Yes/No)” Slow gait speed was determined by analyzing the distribution of walking time in seconds required to advance 3 m starting from a sitting position. Participants in the lowest quintile of this distribution, stratified by height and sex, were classified as having slow gait speed (cutoff point of 0.42 m/s for men and 0.37 for women). Weak handgrip strength was assessed using a dynamometer, with measurements taken from the dominant hand. Participants in the lowest quintile of grip strength, stratified by quartiles of BMI distribution and sex, were identified as having weak handgrip strength (cutoff point of 18.8 kg for men and 11.0 kg for women). To evaluate low physical activity, participants were asked, “Have you engaged in regular exercise or other physically rigorous activities such as sports, jogging, dancing, or heavy work, at least three times a week in the past 12 months? (Yes/No)” Individuals without any of these components were categorized as not frail (robust), those with 1 or 2 components as pre-frail, and those with 3 or more components as frail [40, 41].

### Statistical techniques

For the factor analysis, estimates were derived based on a tetrachoric correlation matrix, enabling the calculation of factor loadings for dichotomous variables (presence/absence). The feasibility of using EFA for identifying possible latent variables was assessed by studying and plotting the correlation between the NCDs at baseline. Additionally, the evaluation incorporated the Kaiser‒Meyer‒Olkin factor adequacy test (KMO = 0.6), and the Bartlett variance homogeneity test (K-squared = 8,257.8; *p* < 2.2e-16). To determine the optimal number of factors for EFA, we assessed eigenvalues, conducted parallel analysis (bootstrap of 100 replications), and an optimal coordinate analysis. Four factors (clusters) were ultimately conserved based on Kaiser criterion (eigenvalue > 1), taking into consideration the etiological sense of the grouping after obtaining the factor values. A comparison with other clustering techniques, using the elbow rule, was conducted to aid in the decision-making process regarding the total number of factors to retain. To obtain factor loadings a maximum likelihood estimation method was used, and a Varimax orthogonal rotation was performed to gain interpretability. The NCD configurations were classified based on the highest factor loading values, and names were assigned relying on an etiological sense between the diseases and physiological systems. Individuals were classified into specific disease configurations based on the factor groups obtained if they met the selected condition of having the diseases included in each factor.

To test the robustness and reproducibility of these configurations, we compared the results from EFA with other clustering techniques, such as simple and multiple correspondence analysis (CA), k-means, fuzzy c-means, and hierarchical clustering. CA took into consideration the variance and contribution of each disease (52%) and were visually assessed with graphical methods. Moving on to fuzzy c-means, the diseases were grouped based on a membership matrix of probabilities. The assignment of diseases to clusters using the K-means technique was determined by evaluating the distances of the NCDs from a centromere. Both for K-means and for hierarchical clustering, the optimal number of clusters was determined by assessing stability using a Jaccard index of 0.75 with 100 bootstrap repetitions (JB).

The decision to employ EFA for identifying latent variables was primarily driven by the interest in exploring the underlying structure of the relationship between variables and uncovering non-observed latent variables. Imputations were performed at the time of death for 60 subjects. This occurred in cases where information about the occurrence of death was available for each follow-up, but the specific date of death was not reported. The procedure for allocating time involved analyzing the distribution of lifespan among those who died. Subsequently, the median lifespan among deceased individuals was determined. Imputed values were 10.1 months (*n* = 16) during the first follow-up and 19.5 months (*n* = 44) during the second.

To assess the association of NCDs and frailty on early mortality, mortality rates by all cause death (per 1,000 person-years) were estimated for age (quinquennial distribution), sex, the accumulation of NCDs and frailty phenotypes. The calculations were made for all the populations (without any stratification), for those without NCDs and stratified by NCD configurations. Then, a Kaplan‒Meier survival analysis was performed to evaluate the death risk of MM, NCD configurations and frailty stratified by sex. A log-rank test was used to assess for significant differences between groups within this analysis.

Models were constructed with a stepwise approach starting from simpler models to examine the relationship between third variables and mortality by all causes. Variables with a potential role as confounders, including various sociodemographic factors, biomarkers, and health-related conditions, were directly selected from the database. The selection was based on their recognized roles in the literature concerning frailty, multimorbidity, and mortality. The variables of sex, age, marital status, C-reactive protein (CRP), educational level, wealth level, cognitive disability according to the Folstein Mini-Mental State Examination (MMSE), and basic functional disability (standardized values) were selected on the basis of contributions to statistical significance of the final models, as assessed by a decrease in the Bayesian Information Criterion (BIC) estimator. To ensure comparability and facilitate interpretation of the COX analyses, the set of covariates contributing to the best statistical models was identified and maintained consistently within the same set for all models.

Cox proportional hazards models [42] were used to analyze the mortality risk for each multimorbidity configuration and frailty phenotype. The influence between the exposure variables and mortality was performed by independently testing each exposure variable in a single adjusted model and then by comparing the HRs when considering adding frailty phenotypes.

Losses to follow-up or subjects who finalized the time in the study without dying were considered right censoring. To verify the proportionality of the risk over time assumption, all models were evaluated by proportional hazards tests and diagnostics based on weighted residuals. Schoenfeld and Martingale residuals were plotted for visual evaluations. The results are presented as hazard ratios (HRs).

A sensitivity analysis was conducted both with and without the imputed values. Despite the low n for imputations, these values draw a decline in the Kaplan-Meier survival curves and contributed to enhancing the precision of the estimates.

All statistical analyses were performed with RStudio for Windows or Stata/IC 15.1 for Windows (64-bit x86-64) software as needed.

## Results

The average age for the whole population at baseline was 76.5 ± 10.4 years, 54% of the population was female, and 87% had basic or lower levels of education (Table 1).

**Table 1:** Overall description of population characteristics during baseline and by distinct disease clusters

n (% [n])	All^*^	NCD configurations (*n* = 2,607)				
		No NCDs	Neuro-Psychiatric	Metabolic	Cardiovascular	Mixt
		236 (9%)^**^	242 (9%)^**^	806 (31%)^**^	296 (11%)^**^	1,027 (39%)^**^
**General characteristics**						
**Age (N)**	2,662	236	242	806	296	1,027
Years	76.5 (± 10.4)	76.2 (± 10.6)	74.8 (± 10.1)	74.3 (± 9.3)	78.4 (± 9.5)	77.2 (± 10.3)
p10 - p90	64–90	63–90	63–87	63–86	65–90	64–90
**Sex**						
**N**	2,662	236	242	806	296	1,027
*Male*	1,217 (46%)	165 (70%)	93 (38%)	422 (52%)	155 (52%)	354 (35%)
*Female*	1,445 (54%)	71 (30%)	149 (62%)	384 (48%)	141 (48%)	673 (66%)
**Marital status**						
**N**	2,653	233	241	805	295	1,026
*No sentimental partner*	1,350 (51%)	109 (47%)	123 (51%)	357 (44%)	154 (52%)	565 (55%)
*Married*	1,303 (49%)	124 (53%)	118 (49%)	448 (56%)	141 (48%)	461 (45%)
**Education**						
**N**	2,662	236	242	806	296	1,027
*Elementary -*	2,315 (87%)	199 (84%)	212 (88%)	693 (86%)	266 (90%)	891 (87%)
*Secondary +*	347 (13%)	37 (16%)	30 (12%)	113 (14%)	30 (10%)	136 (13%)
**Health characteristics**						
**NCD Frequency**						
**N**	2,607	236	242	806	296	1,027
*0*	236 (9%)	100%	-	-	-	-
*1*	564 (22%)	-	51 (21%)	383 (48%)	23 (8%)	107 (10%)
*2*	689 (26%)	-	66 (27%)	315 (39%)	90 (30%)	218 (21%)
*>= 3*	1,118 (43%)	-	125 (52%)	108 (13%)	183 (62%)	702 (68%)
**C-reactive protein**						
**N**	2,463	199	228	758	267	960
*< 10 mg/dl*	2,128 (86%)	178 (89%)	206 (90%)	670 (88%)	236 (88%)	796 (83%)
*>=10 mg/dl*	335 (14%)	21 (11%)	22 (10%)	88 (12%)	31 (12%)	164 (17%)
**Frailty characteristics**						
**Frailty phenotype**						
**N**	2,658	234	241	806	296	1,026
*Robust*	423 (16%)	67 (29%)	29 (12%)	190 (24%)	34 (12%)	103 (10%)
*Pre-frail*	1,786 (67%)	151 (65%)	165 (69%)	531 (66%)	202 (68%)	698 (68%)
*Frail*	449 (17%)	16 (7%)	47 (20%)	85 (11%)	60 (20%)	225 (22%)
**ADL scale** ^**a**^ **(N)**	2,658	233	242	805	296	1,027
Basic disability (0-100)	15.9 (± 26.9)	9.1 (± 23.0)	16.3 (± 28.0)	8.4 (± 17.8)	21.4 (± 31.3)	19.7 (± 28.7)
p10 - p90	0–62	0–25	0–62	0–18	0–78	0–78
**Cognitive scale**^**b**^ **(N)**	2,659	235	242	805	295	1,027
MMSE (0-100)	84.1 (± 12.8)	84.4 (± 13.2)	83.6 (± 12.4)	86.3 (± 11.8)	81.9 (± 13.1)	83.9 (± 13.0)
p10 - p90	64–96	65–96	64–96	68–96	63–96	64–96

* *N* = 2,662 (including losses to follow-up). **: Mean (± SD); n (%)

a: Activity of Daily Living standardized scores; b: Mini-Mental State Examination standardized scores

To evaluate potential dropout bias, baseline characteristics were compared between the follow-up and lost-to-follow-up groups. The follow-up group showed higher age (76.8 vs. 73.6), lower body weight (62.7 vs. 65.2), less hand grip strength (23.0 vs. 24.4), lower wealth level (poor-middle vs. high), lower academic attainment (elementary vs. secondary or more), and a higher prevalence of rural residence (rural vs. urban). However, no differences were observed in the prevalence of frailty phenotypes, multimorbidity, or NCD configurations (Supplementary Table 1).

**Fig. 2:**
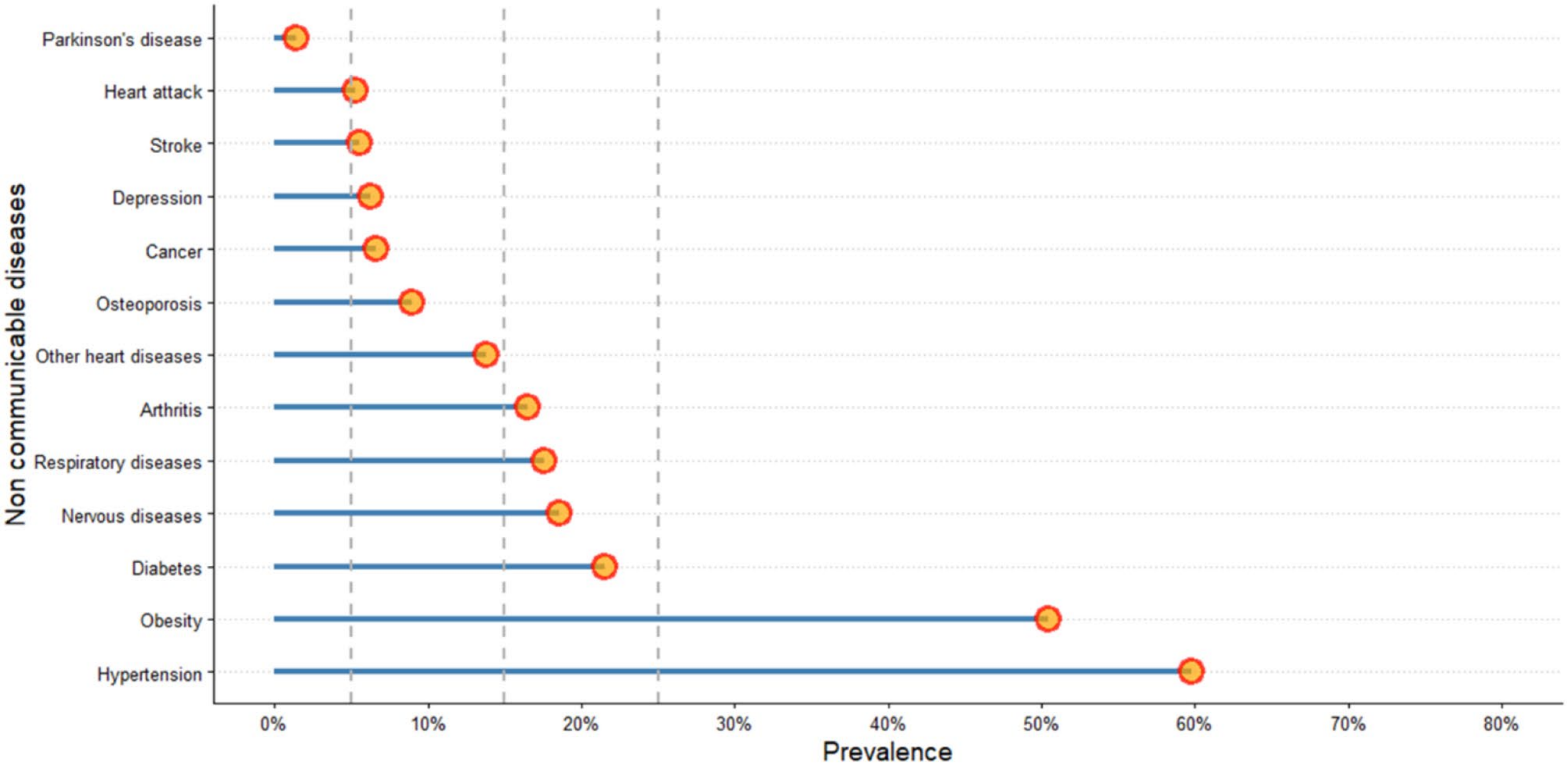
Prevalence of Non-communicable diseases over the basal period (*n* = 2,662)

At baseline, the prevalence of not having any chronic diseases was low and stable with age (∼ 9%), and 22% and 26% of the subjects had 1 and 2 NCDs, respectively (Table 1). When looking solely at the proportions of individuals with a particular disease in Fig. 2, we can see that Parkinson’s disease was low (1.4%; *n* = 38) and hypertension was high (60%; *n* = 1,591) (Fig. 2).

Due to these factors, the co-occurrence of NCDs can be either less or more likely to occur. When comparing 1-on-1 NCDs, the highest value observed was between obesity and HTA (34%) during baseline. For most of the combinations, the prevalence ranged between 1% and 5%, except for obesity and HTA, which concurred with other NCDs, such as DM and HTA (17%), obesity and HTA (15%), nervous diseases and HTA (11.2%) and HTA with other heart diseases (11%), arthritis (11%), and respiratory diseases (10%) (Supplementary Fig. 1).

Almost half of the population (43%) accumulated 3 or more NCDs. Cardiovascular (CVD) and ‘Mixt’ configurations had the largest proportion of individuals with 3 or more diseases (62% and 68%, respectively), followed by ‘Neuro-psychiatric’ (52%) and ‘Metabolic’ (13%) (Table 1).

Four NCD configurations were obtained after the EFA. Obesity, HTA and DM formed the ‘Metabolic’ configuration; heart attack, other heart diseases and stroke formed the ‘CVD’ configuration; chronic pulmonary diseases, arthritis, osteoporosis, and cancer formed the ‘Mixt’ configuration; and chronic nervous problems, depression, and Parkinson’s disease formed the ‘Neuro-psychiatric’ configuration.

Overall, high heterogeneity across cluster conformations was observed with the other techniques, but some patterns could be detected. Obesity, hypertension, and diabetes exhibit consistent patterns when comparing EFA with correspondence analysis, while obesity and hypertension consistently emerge when comparing all techniques. The optimal cluster quantity, regardless of the technique employed, was consistently identified as either three or four (Supplemental Table 2).

**Fig. 3:**
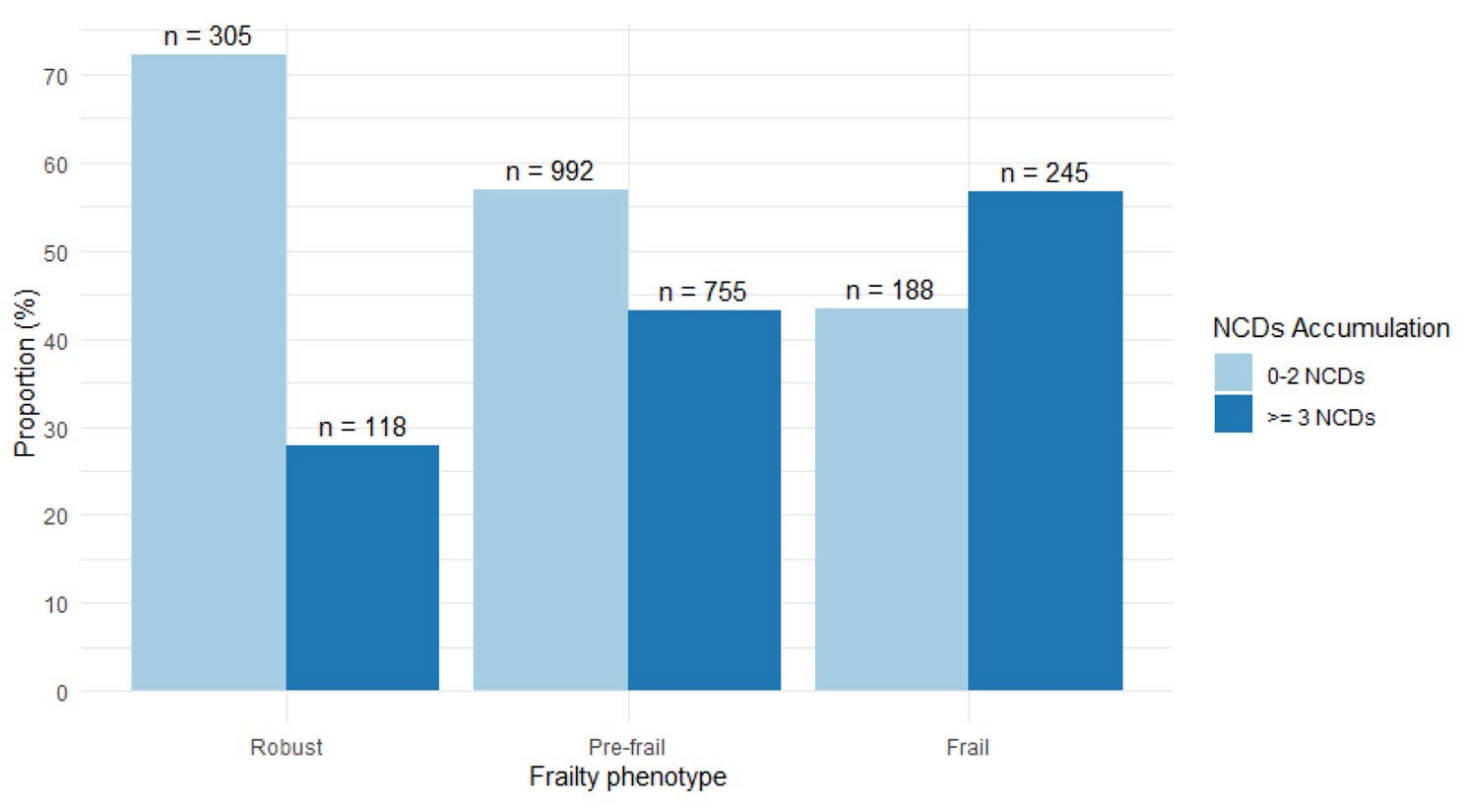
NCD’s accumulation by frailty phenotype (*N* = 2,603)

Frailty subjects showed a higher accumulation of NCDs compared to other phenotypes, constituting 57% of the observed cases. As the cumulative NCD count decreased to two or less, the phenotypes exhibited reduced frailty, as shown in Fig. 3.

Finally, we sought to assess the individual and combined associations of MM, NCD configurations and frailty on early mortality. Overall, mortality rates were higher for individuals in the ‘CVD’ and ‘Mixt’ clusters and were higher with age. These rates increase substantially in subjects from 85 years and older, where the estimations stand out in individuals with at least 90 years old in the ‘Neuro-psychiatric’ and ‘No NCDs’ groups. Mortality is higher when compared to those who have 2 or fewer NCDs, but these rates shift when looking at a specific cluster such as ‘CVD’. Mortality increases as frailty is reached. Within frailty phenotypes, those with frailty who had not reported any NCD had the highest rate (Table 2).

**Table 2:** Description of the different all-cause mortality rates over categories of NCD configurations for multimorbidity

	All	No NCDs	Neuro-Psychiatric	Metabolic	CVD	Mixt
*n*	2,367	216	219	726	279	927
% (n)		9.1	9.3	30.7	11.8	39.2
	Mortality rates *					
**Overall**	71	59	63	44	99	81
**Age (quinquennial)**						
60–64 years	14	-	25	5	27	24
65–69 years	25	-	24	17	33	41
70–74 years	40	47	44	24	88	34
75–79 years	47	36	29	42	53	55
80–84 years	89	83	102	63	111	96
85–89 years	118	61	108	109	154	126
≥ 90 years	292	395	387	235	237	287
**Sex**						
Male	75	55	63	49	92	105
Female	67	68	63	38	107	70
**Frailty phenotype**						
Robust	17	19	44	13	45	9
Pre-frail	65	58	46	45	100	77
Frail	135	258	147	111	131	136
**Accumulation of NCDs**						
0–2 NCDs	64	59	77	45	119	83
≥ 3 NCDs	73	-	51	32	86	80

* Per 1,000 person-years; 566 deaths at the end of follow-up

The Kaplan‒Meier analysis results indicate reduced survival time in frail subjects compared to individuals with a robust or pre-frail phenotype definition (Supplemental Figs. 3 and 5). Survival projections exhibit variability based on the profiles of NCD configurations. Among frail subjects, no differences were observed in survival time across various NCD configurations; however, the group with no reported NCD showed less survival time than any NCD configuration (Supplemental Fig. 3). Higher survival time is observed when assessing mortality with disease accumulation as opposed to NCD configurations (Supplemental Figs. 3 and 4). No significant differences were found in survival time when comparing individuals with 3 or more diseases to those with less than 3 diseases (Supplemental Fig. 4).

Table 3 shows 5 models, each adjusted by the same confounders (see description at the bottom of the table). Each of the models shows the role of distinct chronic NCD configurations in changing frailty risk estimates in the assessed population. The initial three models, presented in the left-to-right columns, depict the independent effects of NCD configurations and frailty on mortality risk. Progressing to the right hand-side of the table, illustrate alterations in mortality risk estimates upon including multimorbidity based on NCD configurations or disease accumulation alongside frailty

**Table 3:** Comparison of frailty and multimorbidity definitions in predicting early mortality: Cox-Regression analyses for disease cluster configurations and frailty phenotypes with 5-Year all-cause mortality (*N* = 2,189)

	NCD configuration only		NCD frequency only	Frailty only	NCD configuration + frailty	NCD frequency + frailty
	HR (95% CI)		HR (95% CI)	HR (95% CI)	HR (95% CI)	HR (95% CI)
**NCD configurations**						
No NCDs	1		-	-	1	-
Neuro-Psychiatric		1.30 (0.80–2.11)	-	-	1.23 (0.76–2.00)	-
Metabolic	1.22 (0.80–1.86)		-	-	1.22 (0.80–1.85)	-
Cardiovascular	1.65 (1.07–2.57) *		-	-	1.56 (1.00–2.42) ^**†**^	-
Mixt	1.49 (1.00–2.22) ^**†**^		-	-	1.42 (0.95–2.11)	-
**NCD Frequency**						
0 to 2 NCDs	-		1	-	-	1
≥ 3 NCDs	-		1.31 (1.09–1.58) **	-	-	1.23 (1.02–1.49) *
**Frailty Phenotype**						
Robust	-		-	1	1	1
Pre-frail	-		-	2.01 (1.29–3.14) **	1.97 (1.26–3.08) **	1.97 (1.26–3.08) **
Frail	-		-	2.45 (1.53–3.93) ***	2.39 (1.48–3.84) ***	2.35 (1.46–3.79) ***

All models were adjusted by sex, age (continuous), civil status (with or without a sentimental partner/married) C-reactive protein (≥ 10 mg/dl), cognitive scale (normalized distribution), education level (≤ basic and ≥ secondary), basic functional disability (normalized distribution) and wealth level (≤ medium/low and ≥ high).^**†**^ *P* = 0.05; * *P* < 0.05; ** *P* < 0.01; *** *P* < 0.001

The ‘Cardiovascular’ and ‘Mixt’ NCD configurations, having more than three NCDs, and the frailty phenotypes, are associated with an elevated mortality risk. However, only frailty phenotypes maintain their heightened risk and statistical significance even after the consideration of NCD configurations or disease accumulation in the models. This observation suggests that frailty phenotypes may play a role in absorbing a portion of the risk when evaluating mortality in the population (Table 3).

In isolated models, ‘Cardiovascular’ and ‘Mixt’ NCD configurations displayed the highest hazards with statistical significance (HR 1.65 [CI 1.07–2.57] and HR 1.49 [CI 1.00–2.22], respectively), surpassing the risk associated with having three or more NCDs alone (HR 1.31 [CI 1.09–1.58]). However, in subsequent models, all NCD configurations lost statistical significance, except for the ‘Cardiovascular’ group, which achieved marginally statistical significance. A similar pattern was observed for the group with more than three NCDs, where statistical significance was attenuated but maintained (Table 3).

### NCD configurations

Frailty is a strong risk factor for mortality, regardless of the presence of any multimorbidity configuration. For frailty phenotypes, the multimorbidity state does not add a significant impact in terms of mortality outcomes, whereas for the disease classifications, the frailty state is important for prognosis (Table 3).

## Discussion

Multimorbidity and NCD configurations were related to frailty, and frailty was evidently related to mortality. When taking both multimorbidity and frailty as predictors of mortality, frailty was the dominant predictor, irrespective of multimorbidity or how NCD configurations were defined. In general, specific configurations, i.e. ‘cardiovascular’ and ‘ mixt’ had a more distinct and higher risk related to mortality than when adding up the number of diseases into an NCD frequency score.

Our study concurs with the findings of a systematic review that explored MM analysis using various clustering methods. In line with our study, the review revealed significant variability in MM patterns depending on the employed methodology. However, the authors of the review also emphasized that the inclusion of different disease types (acute, chronic, or ailments) and the chosen classification approach (categorical, nominal, or continuous) influenced the resulting patterns to some extent. Notably, the review identified three primary groups of MM: cardiovascular and metabolic diseases (such as angina, hypertension, cholesterol, diabetes, edema, and gout); mental health problems (including anxiety, depression, and mood disorders); and allergy-related diseases (such as hay fever, sinusitis, and food allergies) [43].

Chronic diseases have far-reaching impacts across interconnected systems within the human body, surpassing the boundaries of individual systems. The clustering results obtained in this study provide a glimpse into the intricate interdependencies and impacts between diseases and systems. Consequently, relying solely on co-occurrence to assess a patient’s health may fail to capture the complete picture. Adopting comprehensive approaches that consider the dynamic interplay among diseases and systems is indispensable for gaining a more holistic understanding of patient health.

It was found that NCD frequency, NCD configurations and frailty phenotype are related to mortality risk, indicating that there may be a relationship between these two factors. Similar results to those shown in this research were found in another study conducted with a similar analysis and a larger dataset of 92,640 participants but with a different definition of frailty. The researchers determined up to 10 disease configurations to assess for MM and found that the frailty index was an important predictor of mortality regardless of the disease clusters, particularly for those with endocrine, lung, or heart diseases [44].

Different results have been found in other studies, including a cohort study of 7,197 older adults, which identified five different MM patterns with high variability in mortality between frailty states (phenotypes) [28]. The scientific evidence may suggest that regardless of the method used to define the frailty spectrum, MM may be related to promoting changes in these states. A meta-analysis also revealed a bidirectional association between MM and frailty, with 72% (95% IC: 63–81%; I2 = 91.3%) of frail individuals having MM and 16% (95% IC: 12–21%; I2 = 96.5%) of multimorbid individuals being frail [29]. The results obtained from the present analysis revealed that the mortality hazard ratios (HRs) and their statistical significance decreased when frailty phenotypes and MM or any NCD configuration were included together in the Cox model. However, the risk associated with the phenotypes remains relatively stable, regardless of the presence of any NCD type of grouping. Nevertheless, it is essential to acknowledge that these estimates might vary when enhancing the statistical power of the conformed groups.

Definition for MM based on adding diseases may not capture a spectrum of the damage inflicted by specific NCD combinations to the equilibrium in physiological systems [28]. Constant attacks diminish the response capacity, pushing each time more toward a state of fragility and to a consequent collapse. However, certain combinations of chronic diseases may have an effect on hindering responses for recovery [11, 45]. An understanding of this would be very useful to take quick and precise actions before attaining a state of frailty or death. In the present analysis, only chronic diseases were included, which is relevant to the concept of resilience mechanisms [46]. This said, compensation mechanisms could reduce the reserves of system networks in the organism and lead to a state of fragility and consequently to death [29].

One important consideration is the potential influence of ongoing treatments on the development of MM and mortality. In our analysis, receiving pharmacological treatment for each specific NCD when constructing the NCD configurations was part of the definition of the disease. However, it is worth noting that subjects in the study may have also been undergoing a wide variety of alternative treatments, following special diets, or taking supplements that could potentially impact their health status, either through a placebo or a genuine therapeutic response. For future studies on multimorbidity, it could be informative to also take polypharmacy into account.

Given the nature of the study, it possesses certain limitations that should be considered when interpreting the results. First Given the unique life expectancy of this cohort, we cannot guarantee that mortality events will not occur shortly after the last assessment. However, this is less likely to have significantly impacted the results due to Costa Rica’s public health insurance system and the high-quality rating of healthcare, comparable to many developed countries.

The presence for chronic diseases, ADL disabilities, and frailty were constructed using self-report information, relying on self-reported definitions of NCDs and conditions without medical diagnoses during the survey, although self-reporting a diseases assumes a prior diagnosis by a health professional.

Multimorbidity was defined in two distinct ways in this study: as configurations and as the accumulation of diseases. Although the proportion of people with two or more NCDs was highest, indicating the coexistence of morbidities in the configurations, a fraction of the population belonging to a NCD configuration has only 1 chronic disease.

Another aspect to take into consideration is that two of the criteria for determining frailty involve an adaptation based on the availability of information. While an exhaustive exploration of the data was conducted to mitigate potential misclassification, it is essential to emphasize that further statistical validation is required. However, the selected variables are proxies closely aligned with what the model of phenotypes suggests including [38]. One limitation is the question used to assess low activity. It may overestimate the number of people with low activity as some classified as inactive may engage in strenuous activities once or twice a week.

Having considered the above, one advantage of this analysis is the use of a unique nationally representative cohort of Costa Rican older residents [47]. This group underwent comprehensive disease evaluations and detailed phenotyping. In addition to previous reports showing that the association of frailty with mortality is stronger than the association of multimorbidity with mortality in highly developed countries [48–50], the current study confirms this findings in a LMIC with a very different cultural and socioeconomic context.

In this analysis, we utilized EFA and five other clustering techniques to uncover potential latent variables of multimorbidity. However, alternative statistical approaches, such as latent class analysis, may also be suitable for exploring possible latent variables. Additionally, in this analysis, multimorbidity was determined at baseline, but we strongly recommend exploring multimorbidity trajectories to compare the obtained results.

## Conclusions

Frailty accompanying multimorbidity emerges as a more crucial indicator of mortality risk than multimorbidity alone. Therefore, studying NCD configurations is worthwhile as they may offer improved risk profiles for mortality compared to merely using NCD frequency.

### Electronic supplementary material

Below is the link to the electronic supplementary material.

Supplementary Material 1

## Data Availability

“The dataset(s) supporting the conclusions of this article is(are) available in the [National Archive of Computerized Data on Aging (NACDA)] repository, https://www.icpsr.umich.edu/web/NACDA/search/studies?start=0&sort=scoredesc&2CTITLE_SORTasc&ARCHIVE=NACDA&PUBLISH_STATUS=PUBLISHED&rows=50&q=CRELES”.

## Abbreviations

BIC: Bayesian information criterion
BMI: Body mass index
CA: Correspondence analysis
CRELES: Costa Rican Longevity and Healthy Aging Study Cohort
CRP: C-reactive protein
CVD: Cardiovascular
DM: Diabetes
EFA: Exploratory factor analysis
HbA1c: Glycosylated hemoglobin
HRs: Hazard ratios
HTA: Hypertension
JB: Jaccard index with bootstrap repetitions
MM: Multimorbidity
MMSE: Folstein Mini-Mental State Examination
NCD: Noncommunicable disease

## References

[1] Hogan DB. Models, definitions, and criteria for frailty. In: Ram JL, Conn PM, editors. Conn’s handbook of models for human aging. Academic; 2018. pp. 35–44.

[2] Brivio P, Paladini MS, Racagni G, Riva MA, Calabrese F, Molteni R (2019). From healthy aging to Frailty: in search of the underlying mechanisms. Curr Med Chem.

[3] Xia S, Zhang X, Zheng S, Khanabdali R, Kalionis B, Wu J et al. An update on inflamm-aging: mechanisms, prevention, and treatment. J Immunol Res. 2016;2016. 10.1155/2016/8426874 PMC4963991 27493973

[4] Dodig S, Čepelak I, Pavić I. Hallmarks of senescence and aging. Biochem Med (Zagreb). 2019;29(3). 10.11613/BM.2019.030501 PMC6610675 31379458

[5] Kennedy BK, Berger SL, Brunet A, Campisi J, Cuervo AM, Epel ES (2014). Geroscience: linking aging to Chronic Disease. Cell.

[6] Makovski TT, Schmitz S, Zeegers MP, Stranges S, van den Akker M (2019). Multimorbidity and quality of life: systematic literature review and meta-analysis. Ageing Res Rev.

[7] Bloom DE, Boersch-Supan A, Mcgee P, Seike A. Population aging: facts, challenges, and responses. cdn1.sph.harvard.edu. 2011.

[8] Wleklik M, Uchmanowicz I, Jankowska EA, Vitale C, Lisiak M, Drozd M (2020). Multidimensional Approach to Frailty. Front Psychol.

[9] De Biasio JC, Mittel AM, Mueller AL, Ferrante LE, Kim DH, Shaefi S. Frailty in critical care medicine: a review. Anesth Analg. 2020 [cited 2023 Feb 8];130(6):1462. /pmc/articles/PMC7426653/10.1213/ANE.0000000000004665 PMC7426653 32384336

[10] Ferrucci L, Gonzalez-Freire M, Fabbri E, Simonsick E, Tanaka T, Moore Z et al. Measuring biological aging in humans: a quest. Aging Cell. 2020;19(2). 10.1111/acel.13080 PMC6996955 31833194

[11] Skou ST, Mair FS, Fortin M, Guthrie B, Nunes BP, Miranda JJ et al. Multimorbidity. Nat Rev Dis Primers. 2022 [cited 2022 Sep 15];8(1):1–22. https://www.nature.com/articles/s41572-022-00376-4 10.1038/s41572-022-00376-4 PMC7613517 35835758

[12] Le Reste JY, Nabbe P, Manceau B, Lygidakis C, Doerr C, Lingner H (2013). The European General Practice Research Network presents a comprehensive definition of Multimorbidity in Family Medicine and Long Term Care, following a systematic review of relevant Literature. J Am Med Dir Assoc.

[13] Prados-Torres A, Calderón-Larrañaga A, Hancco-Saavedra J, Poblador-Plou B, Van Den Akker M (2014). Multimorbidity patterns: a systematic review. J Clin Epidemiol.

[14] Johnston MC, Crilly M, Black C, Prescott GJ, Mercer SW. Defining and measuring multimorbidity: a systematic review of systematic reviews. Eur J Public Health. 2019 [cited 2022 Oct 5];29(1):182–9. https://academic.oup.com/eurpub/article/29/1/182/5033670 10.1093/eurpub/cky098 29878097

[15] The Academy of Medical Sciences. Multimorbidity: a priority for global health research. 2018. https://acmedsci.ac.uk/policy/policy-projects/multimorbidity

[16] Whitty CJM, Watt FM. Map clusters of diseases to tackle multimorbidity. Nature. 2020 [cited 2023 Dec 18];579(7800):494–6. https://pubmed.ncbi.nlm.nih.gov/32210388/10.1038/d41586-020-00837-4 32210388

[17] Chowdhury SR, Chandra Das D, Sunna TC, Beyene J, Hossain A. Global and regional prevalence of multimorbidity in the adult population in community settings: a systematic review and meta-analysis. EClinicalMedicine. 2023;57. 10.1016/j.eclinm.2023.101860 PMC9971315 36864977

[18] World Health Organization. Noncommunicable diseases country profiles 2018. 2018.

[19] Bennett JE, Stevens GA, Mathers CD, Bonita R, Rehm J, Kruk ME (2018). NCD countdown 2030: worldwide trends in non-communicable disease mortality and progress towards sustainable development goal target 3.4. Lancet.

[20] Kanesarajah J, Waller M, Whitty JA, Mishra GD (2018). Multimorbidity and quality of life at mid-life: a systematic review of general population studies. Maturitas.

[21] Sum G, Salisbury C, Koh GCH, Atun R, Oldenburg B, Mcpake B et al. Implications of multimorbidity patterns on health care utilisation and quality of life in middle-income countries: cross-sectional analysis. J Glob Health. 2019;9(2). 10.7189/jogh.09.020413 PMC6684869 31448114

[22] Ferrer A, Formiga F, Sanz H, Almeda J, Padrós G (2017). Multimorbidity as specific disease combinations, an important predictor factor for mortality in octogenarians: the Octabaix study. Clin Interv Aging.

[23] Kadambi S, Abdallah M, Loh KP (2020). Multimorbidity, function and cognition in aging. Clin Geriatr Med.

[24] Clegg A, Young J, Iliffe S, Rikkert MO, Rockwood K (2013). Frailty in elderly people. Lancet.

[25] Thillainadesan J, Scott IA, Le Couteur DG. Frailty, a multisystem ageing syndrome. Age Ageing. 2020 [cited 2023 Dec 20];49(5):758–63. 10.1093/ageing/afaa112 10.1093/ageing/afaa112 32542377

[26] Rodríguez-Laso Á, Caballero Mora MÁ, García Sánchez I, Alonso Bouzón C, Rodríguez Mañas L, Bernabei R, et al. Updated state of the art report on the prevention and management of frailty. European Union; 2019.

[27] O’Caoimh R, Sezgin D, O’Donovan MR, William Molloy D, Clegg A, Rockwood K (2021). Prevalence of frailty in 62 countries across the world: a systematic review and meta-analysis of population-level studies. Age Ageing.

[28] Nguyen QD, Wu C, Odden MC, Kim DH (2019). Multimorbidity patterns, Frailty, and Survival in Community-Dwelling older adults. Journals Gerontology: Ser A.

[29] Vetrano DL, Palmer K, Marengoni A, Marzetti E, Lattanzio F, Roller-Wirnsberger R et al. Frailty and multimorbidity: a systematic review and meta-analysis. J Gerontol A Biol Sci. 2019 [cited 2020 May 4];74(5):659–66. http://www.ncbi.nlm.nih.gov/pubmed/29726918 10.1093/gerona/gly110 29726918

[30] Juul-Larsen HG, Christensen LD, Bandholm T, Andersen O, Kallemose T, Jørgensen LM (2020). Patterns of Multimorbidity and differences in Healthcare Utilization and complexity among acutely hospitalized medical patients (≥ 65 Years)– A latent class Approach. Clin Epidemiol.

[31] Abizanda P, Romero L, Sanchez-Jurado PM, Martinez-Reig M, Alfonso-Silguero SA, Rodriguez-Manas L. Age, frailty, disability, institutionalization, multimorbidity or comorbidity. Which are the main targets in older adults? Journal of Nutrition, Health and Aging. 2014 [cited 2023 Dec 20];18(6):622–7. https://link.springer.com/article/10.1007/s12603-014-0033-310.1007/s12603-014-0033-324950154

[32] Abizanda P, Rodríguez-Mañas L. Function but not multimorbidity at the cornerstone of geriatric medicine. J Am Geriatr Soc. 2017 [cited 2023 Dec 20];65(10):2333–4. https://pubmed.ncbi.nlm.nih.gov/28832930/10.1111/jgs.15021 28832930

[33] Vetrano DL, Calderón-Larrañaga A, Marengoni A, Onder G, Bauer JM, Cesari M (2018). An International Perspective on Chronic Multimorbidity: approaching the Elephant in the room. Journals Gerontology: Ser A.

[34] Rosero-Bixby L, Fernández X, Dow WH. CRELES: Costa Rican longevity and healthy aging study, 2005 (Costa Rica Estudio de Longevidad y Envejecimiento Saludable). Inter-university Consortium for Political and Social Research [distributor]. NACDA: National Archive of Computerized Data on Aging; 2013 [cited 2023 Aug 24]. https://repository.synchros.eu/study/creles

[35] Méndez Chacón E, Rosero Bixby L (2007). Prevalencia De hipertensión en adultos mayores de Costa Rica. Poblac Salud Mesoam.

[36] Brenes Camacho G, Rosero-Bixby L. Diabetes mellitus en adultos mayores costarricenses. Poblac Salud Mesoam. 2007;5(1).

[37] Burki T (2021). European Commission classifies obesity as a chronic disease. Lancet Diabetes Endocrinol.

[38] Rosero-Bixby L, Dow WH, Brenes G. Costa Rican longevity and healthy aging study. Encyclopedia Gerontol Popul Aging. 2019:1–5.

[39] Alden D, Austin C, Sturgeon R (1989). A correlation between the geriatric Depression Scale Long and short forms. J Gerontol.

[40] Fried LP, Tangen CM, Walston J, Newman AB, Hirsch C, Gottdiener J (2001). Frailty in older adults: evidence for a phenotype. J Gerontol Biol Sci Med Sci.

[41] Rosero Bixby L, Dow WH, Brenes Camacho G. Costa Rican longevity and healthy aging study. 2019.

[42] Gómez Melis G, Cadarso-Suárez C (2017). El Modelo De Riesgos Proporcionales De Cox Y sus extensiones. Impacto en estadística y biomedicina - dialnet. Gaceta De La Real Sociedad Matemática Española.

[43] Ng SK, Tawiah R, Sawyer M, Scuffham P (2018). Patterns of multimorbid health conditions: a systematic review of analytical methods and comparison analysis. Int J Epidemiol.

[44] Oude Voshaar RC, Jeuring HW, Borges MK, van den Brink RHS, Marijnissen RM, Hoogendijk EO et al. Course of frailty stratified by physical and mental multimorbidity patterns: a 5-year follow-up of 92,640 participants of the lifelines cohort study. BMC Med. 2021;19(1). 10.1186/s12916-021-01904-x PMC7869455 33550989

[45] MacRae C, McMinn M, Mercer SW, Henderson D, McAllister DA, Ho I et al. The impact of varying the number and selection of conditions on estimated multimorbidity prevalence: a cross-sectional study using a large, primary care population dataset. PLoS Med. 2023 [cited 2024 Jan 17];20(4). https://pubmed.ncbi.nlm.nih.gov/37014910/10.1371/journal.pmed.1004208 PMC10072475 37014910

[46] Wister AV, Multimorbidity Resilience. Conceptual, theoretical, and measurement developments. Resil Aging. 2020:81–105.

[47] Rosero-Bixby L. The exceptionally high life expectancy of Costa Rican nonagenarians. Demography. 2008 [cited 2024 Jan 4];45(3):673. /pmc/articles/PMC2831395/10.1353/dem.0.0011 PMC2831395 18939667

[48] Rosero-Bixby L, Dow WH. Exploring why Costa Rica outperforms the United States in life expectancy: a tale of two inequality gradients. P Natl A Sci. 2016 [cited 2024 Jan 4];113(5):1130–7. https://www.pnas.org/doi/abs/10.1073/pnas.1521917112 10.1073/pnas.1521917112 PMC4747769 26729886

[49] Rosero-Bixby L, Dow WH (2009). Surprising SES gradients in mortality, health, and biomarkers in a latin American population of adults. J Gerontol B Psychol Sci Soc Sci.

[50] Basto-Abreu A, Barrientos-Gutierrez T, Wade AN, de Melo DO, de Souza ASS, Nunes BP et al. Multimorbidity matters in low and middle-income countries. 10.1177/26335565221106074. 2022 [cited 2024 Jan 17];12:263355652211060. https://journals.sagepub.com/doi/full/10.1177/26335565221106074 10.1177/26335565221106074 PMC9208045 35734547

