# Supplementary material for: Assessing the relationship between multimorbidity, NCD configurations, frailty phenotypes, and mortality risk in older adults
Source: BMC Geriatr. 2024 Apr 22;24:355. doi: 10.1186/s12877-024-04948-9 (PMC11034053; doi:10.1186/s12877-024-04948-9)

**Supplemental Table 1**. Follow-up Vs Lost to follow-up: Population characteristics at baseline (n = 2,662)

| Characteristic | Follow-up, n = 2,421 (91%)^1^ | Lost to follow-up, n = 241 (9.1%)^1^ | p-value^2^ |
| --- | --- | --- | --- |
| **Age (years)** | 76.8; (10.5) | 73.6; (8.4) | **<0.001** |
| **Weight (Kg)** | 62.7; (14.0) | 65.2; (13.9) | **0.007** |
| **Height (cm)** | 154.9; (9.6) | 155.8; (9.8) | 0.2 |
| **BMI (kg/m^2)** | 25.9; (4.9) | 26.8; (4.9) | **0.013** |
| **Grip strength (kg)** | 23.0; (8.7) | 24.4; (8.1) | **0.016** |
| **Gait speed (mts/s)** | 0.6; (0.2) | 0.6; (0.2) | 0.2 |
| **Sex** |  |  | 0.4 |
| *Male* | 1,101/2,421 (45%) | 116/241 (48%) |  |
| *Female* | 1,320/2,421 (55%) | 125/241 (52%) |  |
| **Wealth** |  |  | **0.030** |
| *Poor-middle* | 2,203/2,421 (91%) | 209/241 (87%) |  |
| *High* | 218/2,421 (9.0%) | 32/241 (13%) |  |
| **Education** |  |  | **<0.001** |
| *Elementary -* | 2,126/2,421 (88%) | 189/241 (78%) |  |
| *Secondary +* | 295/2,421 (12%) | 52/241 (22%) |  |
| **Civil status** |  |  | 0.9 |
| *No sentimental partner* | 1,226/2,412 (51%) | 124/241 (51%) |  |
| *Married* | 1,186/2,412 (49%) | 117/241 (49%) |  |
| **Living in Urban** |  |  | **<0.001** |
| *Rural* | 1,008/2,421 (42%) | 74/241 (31%) |  |
| *Urban* | 1,413/2,421 (58%) | 167/241 (69%) |  |
| **Frailty phenotypes** |  |  | 0.12 |
| *Robust* | 377/2,363 (16%) | 46/240 (19%) |  |
| *Pre-frail* | 1,583/2,363 (67%) | 164/240 (68%) |  |
| *Frail* | 403/2,363 (17%) | 30/240 (12%) |  |
| **NCD configurations** |  |  | 0.3 |
| *No NCDs* | 216/2,367 (9.1%) | 20/240 (8.3%) |  |
| *Neuro-Psychiatric* | 219/2,367 (9.3%) | 23/240 (9.6%) |  |
| *Metabolic* | 726/2,367 (31%) | 80/240 (33%) |  |
| *Cardiovascular* | 279/2,367 (12%) | 17/240 (7.1%) |  |
| *Mixt* | 927/2,367 (39%) | 100/240 (42%) |  |
| **Disease accumulation** |  |  | 0.2 |
| *0-2 NCDs* | 1,361/2,367 (57%) | 128/240 (53%) |  |
| *≥ 3 NCDs* | 1,006/2,367 (43%) | 112/240 (47%) |  |
| ^1^Mean; (SD); n/N (%) | | | |
| ^2^Wilcoxon rank sum test; Pearson's Chi-squared test | | | |

**Supplemental Figure 1.** Heatmap for prevalence and co-occurrence patterns for non-communicable diseases during baseline (n=2,662)

**
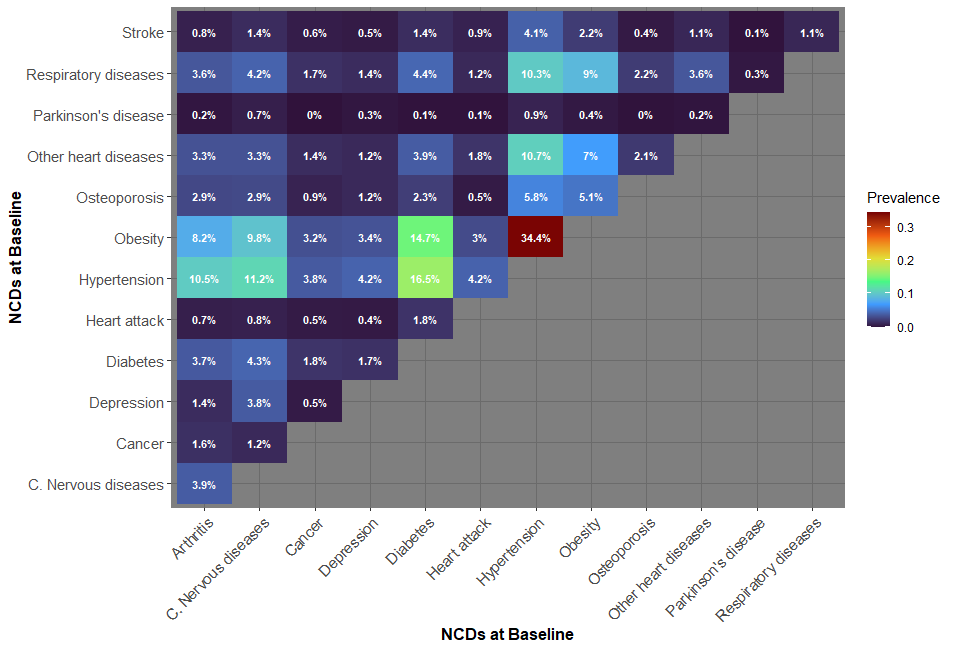
**

**Supplemental Figure 2**. Exploratory factorial analysis and Clustering analysis results for chronic NCD configurations at baseline.

* Visual reference; ** F stands for factor and C stands for cluster. **a** Dendrogram shows a possible 4th aggrupation. **b**: metabolic; **c**: cardiovascular; **d**: mixt; **e**: neuro psychiatric.

**Supplemental Figure 3**: Survival in months by NCDs configurations and frailty phenotype’s


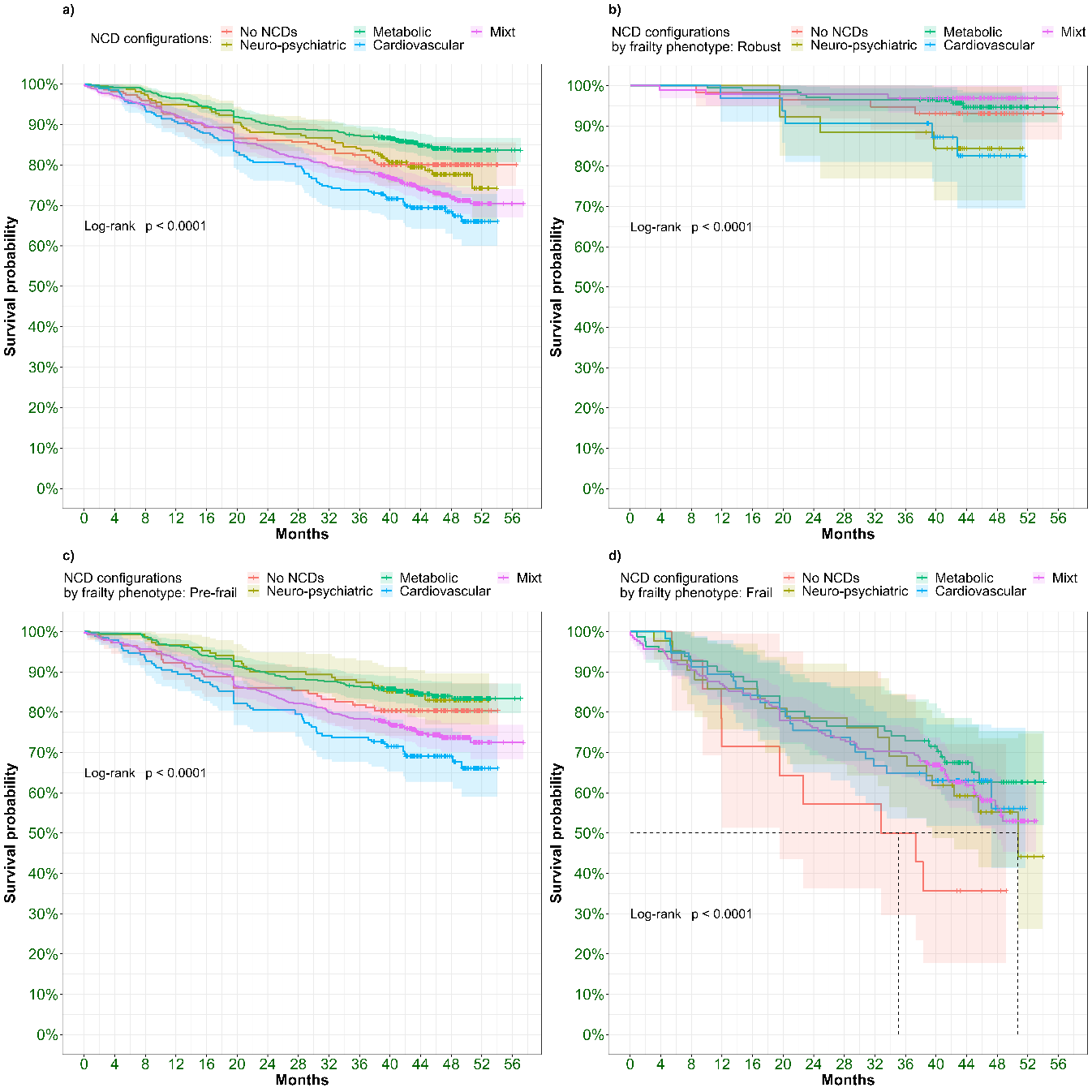


**Supplemental Figure 4**: Survival in months by NCDs accumulation


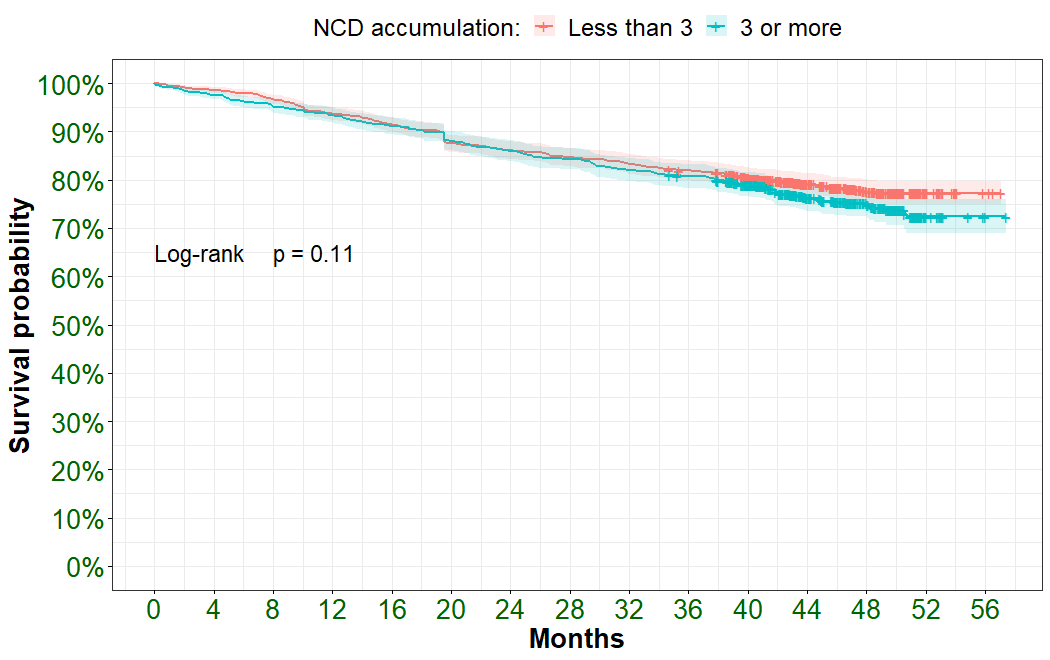


**Supplemental Figure 5**: Survival in months by frailty phenotype and sex


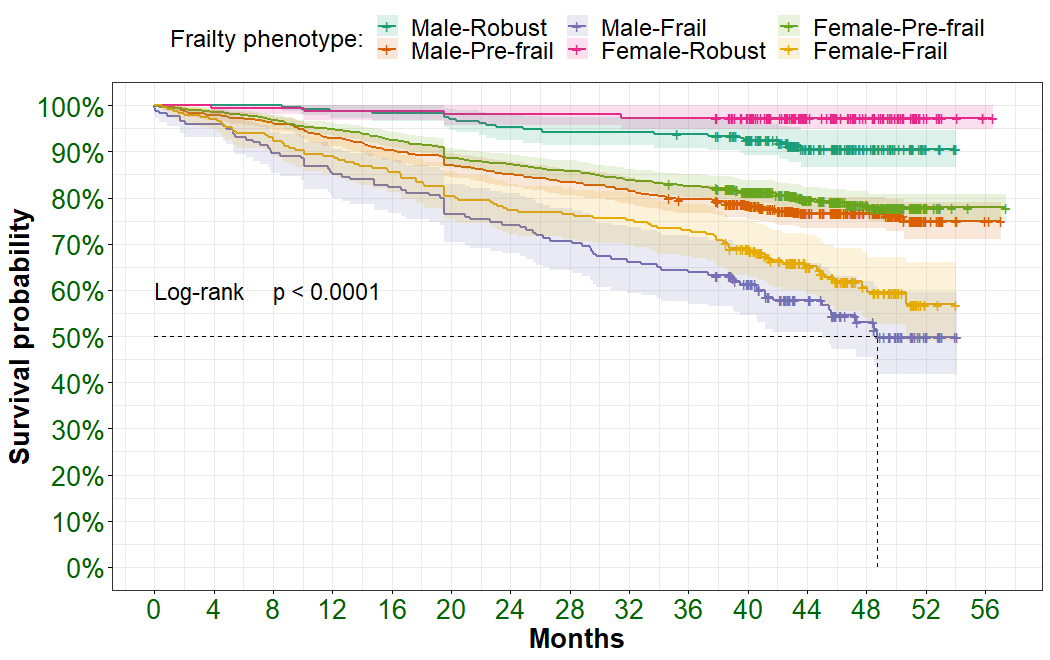

Supplement: Supplementary file 1 — Supplementary Material 1 [file 12877_2024_4948_MOESM1_ESM.docx]
